# Cistrome: an integrative platform for transcriptional regulation studies

**DOI:** 10.1186/gb-2011-12-8-r83

**Published:** 2011-08-22

**Authors:** Tao Liu, Jorge A Ortiz, Len Taing, Clifford A Meyer, Bernett Lee, Yong Zhang, Hyunjin Shin, Swee S Wong, Jian Ma, Ying Lei, Utz J Pape, Michael Poidinger, Yiwen Chen, Kevin Yeung, Myles Brown, Yaron Turpaz, X Shirley Liu

**Affiliations:** 1 Department of Biostatistics and Computational Biology, Dana-Farber Cancer Institute and Harvard School of Public Health, 450 Brookline Ave, Boston, MA 02215, USA; 2 Center for Functional Cancer Epigenetics, Dana-Farber Cancer Institute, Boston, MA 02215, USA; 3 Lilly Singapore Centre for Drug Discovery, 8A Biomedical Grove, Immunos, Singapore 138648; 4 Beijing Genomics Institute, Beishan Industrial Zone, Yantian District, Shenzhen 518083, China; 5 Singapore Immunology Network, 8A Biomedical Grove, Immunos Building level 3, Singapore 138648; 6 School of Life Science and Technology, Tongji University, 1239 Siping Road, Shanghai 200092, China; 7 Eli Lilly and Company, Lilly Corporate Center, Indianapolis, IN 46285, USA; 8 Department of Bioengineering, Stanford University, 318 Campus Drive, Stanford, CA 94305, USA; 9 Jardine Lloyd Thompson Asia, 1 Raffles Quay #27-01, One Raffles Quay - North Tower, Singapore 048583; 10 Department of Medical Oncology, Dana-Farber Cancer Institute and Harvard Medical School, 450 Brookline Ave, Boston, MA 02215, USA; 11 AstraZeneca Pharmaceuticals LP, 35 Gatehouse Drive, Waltham, MA 02451, USA

## Abstract

The increasing volume of ChIP-chip and ChIP-seq data being generated creates a challenge for standard, integrative and reproducible bioinformatics data analysis platforms. We developed a web-based application called Cistrome, based on the Galaxy open source framework. In addition to the standard Galaxy functions, Cistrome has 29 ChIP-chip- and ChIP-seq-specific tools in three major categories, from preliminary peak calling and correlation analyses to downstream genome feature association, gene expression analyses, and motif discovery. Cistrome is available at http://cistrome.org/ap/.

## Rationale

The term 'cistrome' refers to the set of *cis*-acting targets of a *trans*-acting factor on a genome-wide scale, also known as the *in vivo *genome-wide location of transcription factors or histone modifications. Cistromes were initially identified using chromatin immunoprecipitation (ChIP) combined with microarrays (ChIP-chip) [1]. However, with the recent advent of next generation sequencing (NGS) technologies, ChIP combined with NGS (ChIP-seq) [2] has become the more popular technique due to its higher sensitivity and resolution.

Computational analyses of cistrome data have become increasingly complex and integrative. Investigators often examine the data from many different angles by combining cistrome, epigenome, genomic sequence, and transcriptome analyses. Many algorithms and tools have been published over the years to facilitate such analyses. However, these tools require investigators to have both the hardware resources and computational expertise to install, configure, and run these different algorithms effectively. Integrated platforms such as CisGenome [3] and seqMINER [4] have been developed to streamline data analyses; however, the maintenance of these platforms demands suitable hardware resources and computational skills. In addition, these tools lack useful features such as the integration of cistrome data with gene expression analysis, data sharing between researchers, and reusable analysis workflows.

**Figure 1:**
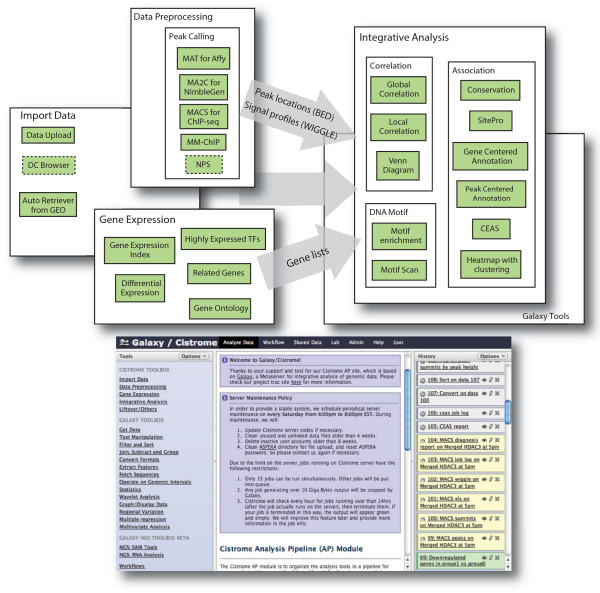
**Workflow within the Cistrome analysis platform**. Cistrome functions can be divided into three categories: data preprocessing, gene expression and integrative analysis. A general workflow using Cistrome is to upload datasets, preprocess them using peak calling tools to generate peak locations in BED format and signal profiles in WIGGLE format, upload gene expression data to produce specific gene lists, and then use various integrative analysis tools to generate figures and reports. The bottom figure shows the web interface of the Cistrome platform based on the Galaxy framework. The left panel shows available tools, the middle panel shows messages, tool options, or result details, and the right panel shows the datasets organized in the user's history, including datasets that have been or are being processed (in green and yellow, respectively), or waiting in the queue (in gray). CEAS,; DC, Data Collection module; GEO, Gene Expression Omnibus; NPS, Nucleosome Positioning from Sequencing; TF, transcription factor.

To address the above challenges, we developed the Cistrome platform to provide a flexible bioinformatics workbench with an analysis platform for ChIP-chip/seq and gene expression microarray analysis. Cistrome was built on top of Galaxy [5], an open-source web based computational framework that allows the easy integration of different tools. Cistrome integrates useful functions specific for ChIP-chip/seq and gene expression analyses. These functions were implemented in a modular fashion to allow easy incorporation of new tools in the future. Cistrome was deployed on a supercomputer server with a publicly available web interface. The current Cistrome server allows 15 jobs running at the same time. Restrictions of input files for each Cistrome tool are described in Table S1 in Additional file 1. We provide Cistrome source codes freely available through bitbucket [6]. The various functions within the analysis platform are explained in the following sections, and a workflow summary is illustrated in Figure 1.

## Data preprocessing

Before interpreting the biological results from ChIP-chip or ChIP-seq data using the Cistrome platform, researchers can upload raw data from their microarray or sequencing facilities and then preprocess those data using Cistrome peak-calling tools. Alternatively, researchers can also upload intermediate results from their own analysis tools. As illustrated in Figure 1, the peak calling step generates two types of intermediate files: peak location files (in BED format), indicating the predicted transcription factor binding sites or histone modification sites, and signal profile files (in WIGGLE format) of binding or histone modification across the genome.

Several methods can be used to import data into Cistrome. The 'Upload File' function can import a file from the user's computer or from an HTTP or FTP file server in the same manner as in Galaxy. In most cases, sequencing facilities will manage the low level base calling and read mapping processes. The least processed Cistrome data formats that we allow are the SAM/BAM [7] or BED formats for ChIP-seq sequencing mapping results, CEL files for ChIP-chip using Affymetrix tiling arrays, or PAIR files from NimbleGen custom arrays. Researchers may have already used other algorithms to generate intermediate results, such as BED format files for regions of interest on the genome or WIGGLE format files for signal information. In such cases, users can also upload intermediate result files onto Cistrome and apply our downstream tools while being mindful of the acceptable formats (Table S1 in Additional file 1). In addition, we implemented two new data types for expression microarray data sets from Affymetrix and NimbleGen technologies. Raw expression microarray data and a text file describing the phenotype information (for example, before and after transcription factor activation) should be packaged in a zip file before being uploaded through the general upload tool.

Cistrome contains peak-calling tools for both ChIP-chip and ChIP-seq data. We deployed the MAT tool [8] for Affymetrix promoter or tiling arrays and have supported nine different array designs from *Caenorhabditis elegans *to human. Affymetrix CEL files are required as input. For NimbleGen two-color arrays, MA2C [9] was deployed. Because researchers usually have their own customized NimbleGen two-color array designs, array design (.ndf) and position (.pos) files and raw probe raw signal files (.pair) should all be uploaded to run MA2C on the Cistrome website. Both MAT and MA2C are able to handle control data or replicates as input data and can generate a BED file for peak locations and WIGGLE file for normalized probe signals as the output. Cistrome provides the MACS (Model-based Analysis of ChIP-Seq) [10] tool for ChIP-seq data obtained from various short read sequencers (for example, Genome Analyzer and HiSeq 2000 from Illumina or SOLiD from Applied Biosystems). MACS can improve the accuracy of the predicted binding sites by modeling the length of the sequenced ChIP fragments and the local bias due to chromatin openness. MACS can run with or without controls and allows the widely used SAM/BAM format and another six mapping result formats (Table S1 in Additional file 1) as input. The outputs include peak regions and peak summits (the precise binding location estimated by the algorithm) in BED format and ChIP fragment pileup along the whole genome at every 10 bp in WIGGLE format. When the diagnosis option is turned on, MACS subsamples the data to determine the number of peaks that can be recovered from a subset, thus estimating the saturation status of the current sequencing depth. We deployed MACS version 1.4rc2 on Cistrome, which supports single-end or paired-end sequencing in BAM or SAM format.

With the rapid growth of ChIP-chip and ChIP-seq datasets in public repositories, it has become increasingly important to be able to integrate information from cross-platform and between-laboratory ChIP-chip or ChIP-seq datasets. We recently developed the powerful meta-analysis tool MM-ChIP (Model-based Meta-analysis of ChIP data) [11] and deployed it under the peak-caller application category of Cistrome. The MM-ChIP tool includes two separate functions: MMChIP-chip performs ChIP-chip meta-analysis based on WIGGLE files from the MA2C and MAT tools, and MMChIP-seq uses NGS alignments in BED format as input to combine different ChIP-seq libraries of the same factor under the same conditions. The resulting peak locations (in BED files) and signal profiles (in WIGGLE files) can be visualized as a custom track on the UCSC genome browser and used as input for other downstream analysis tools that will be discussed later. In addition to these specific peak callers for different platforms or purposes, there is a general peak caller in Cistrome that can take any whole genome signal profile in WIGGLE format, normalize the signals, and then attempt to find the significant regions by comparing to a null distribution built from background data.

## Expression microarray analysis tools

The Cistrome Expression pipeline uses R and Bioconductor [12] packages to perform basic gene expression analyses. The data analysis starts with the processing of a set of signal intensity files for Affymetrix expression arrays (.cel) or NimbleGen arrays (.xys). Datasets may also include a phenotype (.txt) file that describes and groups the set of expression files. The next step in the pipeline calculates the expression index of this dataset using one of four possible methods: robust multichip average (RMA) [13], justRMA, gcRMA and MAS5. The result is a normalized expression set (.eset) that can be represented as refSeq, Entrez, or ProbeSet IDs in plain text format. When mapping the ProbeSet IDs to refSeq or Entrez IDs, the custom CDF files from BRAINARRAY [14] are used. The genes that are differentially expressed between conditions (for example, before and after a transcription factor is knocked down) are often used to explore the function of the transcription factor together with cistrome data. When a normalized expression set is used as input, Cistrome can identify differentially expressed genes using any of the following methods: limma moderated *t*-test, ordinary least-squares, and permutation by re-sampling. Correction for false positive (type I) errors may be performed using either the Bonferroni correction or Benjamini-Hochberg false discovery rate (FDR) methods. The output from this tool is a list of differentially expressed genes, log2-transformed fold changes and FDR-corrected *P*-values of differential expression. The differential expression result can be processed into gene lists, such as up-regulated or down-regulated genes, using one of the public workflows as described in Table S2 in Additional file 1. The gene lists can be further incorporated with other Cistrome tools.

Several downstream analysis modules are also available. A transcription factor tool allows the user to find the transcription factors with the highest level of expression. The selection is done based on an expression index cutoff value, and further filtering can be performed to restrict the resulting list to the Gene Ontology (GO) terms for transcription regulation activities. A correlation tool allows the user to detect all genes for which their expressions correlate with another given gene. This correlation result can also be filtered by applying the GO terms. The GO enrichment tool helps researchers explore the functions for a list of genes, such as the up-regulated genes after a transcription factor knockdown or the genes with transcription factor bound in promoter regions. Enrichment can be compared to the background of all genes or a subset of genes on the array. This tool uses Bioconductor GO and GOstats [15] packages together with a query to the DAVID (Database for Annotation, Visualization and Integrated Discovery) web server [16]. The visualization tool in this category allows users to visualize and compare the expression index distributions of multiple lists of genes (for example, genes with proximate transcription factor binding compared with all genes) using box plots or histograms.

## Integrative analysis

Downstream analyses for a cistrome study require specific or integrative tools. The value of Cistrome is that it enables biologists to use a broad range of bioinformatics tools to easily generate report-quality figures and tables, and to simplify routine analysis using reproducible pipelines. In Cistrome, we provide tools for correlation studies, genome feature association studies and motif analysis together with public workflows to link these tools together.

**Figure 2:**
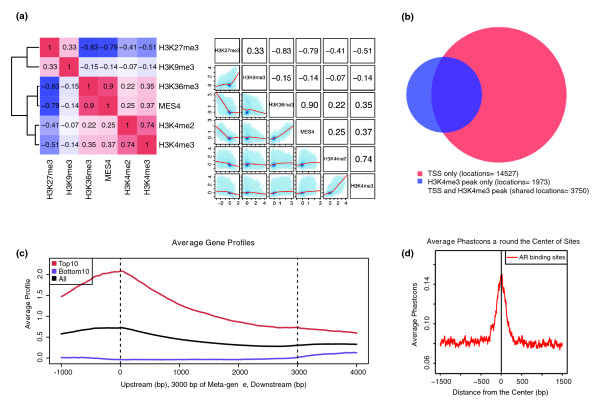
**Correlation and association tools**. **(a) **Correlation plots using different histone marks in *C. elegans *early embryos [43]. Cistrome correlation tools can generate either a heatmap with hierarchical clustering according to pair-wise correlation coefficients or a grid of scatterplots. **(b) **Venn diagram showing the overlap of H3K4me3 peaks (in blue) with transcription start sites (TSS) for all the genes (in red) in the *C. elegans *genome. **(c) **Meta-gene plot generated by CEAS showing the H3K4me3 signals enriched at gene promoter regions; the top expressed genes (red) have higher H3K4me3 signals than the bottom expressed genes (purple). **(d) **Conservation plot showing that the human androgen receptor (AR) binding sites from ChIP-chip [24] are more conserved than their flanking regions in placental mammals.

Usually, researchers require at least two biological replicates to show the consistency of an experiment. An intuitive way to show consistency is to ask if the replicates can be correlated in some meaningful measurement. Correlation can also answer the question of whether or not two transcription factors are co-localized. For instance, two biological replicates with low correlation might suggest poor data quality, or highly overlapping cistromes between two factors might suggest interactions between the factors. For these reasons, we deployed two levels of tools in Cistrome to calculate correlations: one to compare protein-DNA binding signals and the other to investigate the overlap of the predicted binding sites. First, Cistrome can calculate Pearson correlation coefficients for multiple signal profiles on a whole-genome scale or by restricting the calculation to a set of genomic regions defined by the user. A Pearson correlation coefficient close to 1 implies that the replicates are consistent or two factors are correlated. To save computation time, these tools use window-smoothing methods to calculate the mean or median values within non-overlapping fixed-size windows. This approach decreases the number of data points involved in the calculation. The results are represented as scatter plots or heatmap images in either PDF or PNG format as illustrated in Figure 2a. The second level of correlation can address how many of the predicted binding sites (peaks) from several replicates, different factors or different conditions overlap. We provide a tool for drawing a Venn diagram using two to three BED format peak files. The circles and overlapping regions in the Venn diagram can be proportional to the actual number of peaks and overlaps (Figure 2b).

Functional DNA regions in genomes are often evolutionarily conserved between different species [17-19]. Therefore, evolutionary conservation of ChIP-chip/seq peaks compared with flanking non-peak regions is often a good indicator of good data quality and correct data preprocessing. In Cistrome, the 'Conservation Plot' tool can take one or more cistromes in BED files as input, and use UCSC PhastCons conservation scores [20] to produce a figure showing the average conservation score profiles around the peak centers (Figure 2d). This analysis could be extended to compare the conservation differences between multiple cistromes.

Another useful task is to find the genomic features or genes associated with transcription factor binding or histone modification sites. For instance, H3K4me3 is enriched in the promoter regions of active genes [21], and H3K36me3 is enriched in transcribed exons [22]. Finding the target genes is critical to understanding the function of transcription factors, such as transcription repression or activation. Therefore, a set of tools from the CEAS (Cis-regulatory Element Annotation System) [23] package, including SitePro, GCA (Gene Centered Annotation), Peak2Gene and the CEAS main program, has been deployed in the Cistrome web interface. SitePro can draw the average signal profiles around given genomic locations. When multiple locations or sets of signal files are used as input, SitePro can address questions such as how the signals of multiple factors change at the same locations between different conditions or how the same factor changes in different sets of genomic locations. The GCA tool can find the peaks that are closest to the transcription start site (TSS) of each gene and calculate the coverage of the peaks of the gene body in a spreadsheet. The Peak2Gene tool can find the nearest genes for each peak. The CEAS main program generates multi-paged figures as either a PDF document or PNG image. In general, when a BED file for peaks and a WIGGLE file for signals are used as input, the resulting report includes the peak enrichment on chromosomes and various genomic features, such as gene promoters, downstream regions, UTRs, coding exons or introns, and the average signal profile around TSSs and transcription termination sites (TTSs), the meta-gene body (all genes are scaled to 3 kbps), concatenated exons (coding regions), or concatenated introns. When gene lists are provided (for example, a list of genes with the highest and lowest levels of expression for the same sample in a ChIP-chip or ChIP-seq experiment), CEAS will plot the average signal profiles for different gene groups in different colors for the TSS, TTS, gene bodies, exons, or introns (Figure 2c). This function can be coupled with gene expression tools described in the previous section to show whether the signals of the transcription factor or histone marks are related to transcription repression or activation.

**Figure 3:**
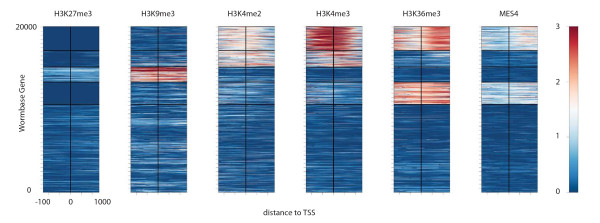
**Heatmap analysis with k-means clustering**. By combining H3K27me3, H3K9me3, H3K4me3, H3K4me2, H3K36me3 and MES-4 (the histone H3K36 methyltransferase) ChIP-chip signals, as in Figure 2a, the Cistrome heatmap tool separates the ± 1-kbp regions for all of the *C. elegans *TSSs into five clusters using k-means clustering. From top to bottom, the clusters are as follows: (1) about 3,000 TSSs related to active genes have high H3K4me3 upstream of the TSSs and high H3K36me3 downstream of the TSSs; (2) about 2,000 TTSs have slightly lower H3K4me3 levels downstream of the TSSs and no significant K36me3 enrichment; (3) about 2,000 TSSs have high H3K27me3 and H3K9me3 related to inactive genes; (4) about 2,500 TTSs with low H3K27me3, moderate H3K4me3 and high H3K36me3 enrichment around the TTS related to genes in operons; and (5) about 10,000 TTSs have no strong marks.

In addition to the average signal profiles at a given set of genomic locations, as shown in CEAS, the visualization and clustering of signal profiles from different factors at specific locations provides another angle of insight. Through the observation of patterns, we can also find the co-factors (co-activators or co-repressors) that tend to work together on their regulated genes. The Cistrome 'Heatmap' tool can extract the signals centered at every given genomic location, perform either a k-means clustering or a sorting by maximum, mean, or median values within each region, and then draw a heatmap. For example, the group of TSSs for active genes should have H3K4me3 enriched at the TSS and a gradual H3K36me3 enrichment downstream of the TSS, whereas the group of TSSs for inactive genes would have low signals of both H3K4me3 and H3K36me3. Additional detailed clustering will be revealed when signal profiles of multiple factors are used (Figure 3). Multiple WIGGLE files for different factors or different conditions can be used as input together with a set of genomic locations defined in a BED file. These regions could be nucleosome-free regions or transcription factor binding sites instead of TSSs of genes. Clustering or sorting can be based on all or some of the WIGGLE files. The color schema of the heatmap is configurable to adjust the contrast for better visualization between high and low signals.

**Figure 4:**
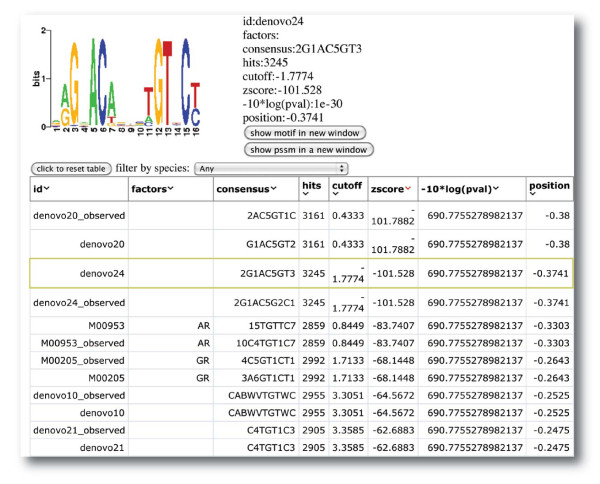
**Cistrome SeqPos motif analysis**. A screenshot of the SeqPos output. The enriched motifs at the androgen receptor binding sites without FoxA1 binding are displayed in an interactive HTML page. When the user clicks on the row of a particular motif, the motif logo and detail information are shown at the top of the page.

Transcription factor motif analysis is a key to understand the specific DNA patterns of *in vivo *transcription factor binding. Motif analysis can also identify the co-factors that work together to activate or repress gene expression because the binding sites of co-factors should have similar DNA motifs. We deployed a new motif algorithm called 'SeqPos' in Cistrome based on the algorithm in [24]. By taking the peak locations as the input, SeqPos can find motifs that are enriched close to the peak centers. SeqPos can scan all of the motifs that we collected from JASPAR [25], TRANSFAC [26], Protein Binding Microarray (PBM) [27], Yeast-1-hybrid (y1h) [28], and the human protein-DNA interaction (hPDI) databases [29]. SeqPos can also find *de novo *motifs using the MDscan algorithm [30]. The final significant motifs are listed in an HTML page, as in Figure 4, where the user can sort the motifs by z-score or *P*-value and click on each motif to see detailed information, such as the probability matrix, logos, and the motif consensus. A position-specific scoring matrix can be copied or referred to another tool within Cistrome called a 'screen motif' to search a given set of genomic locations for all occurrences of a particular motif.

Cistrome has many other useful tools to help users better manipulate their data. A lift over tool can convert WIGGLE files from one genome assembly to another if users want to combine old analysis results with a new genome annotation. However, *ab initio *re-preprocessing is recommended to generate new WIGGLE files for the new genome assembly. A WIGGLE file standardization tool can convert the resolution of a WIGGLE file to 8, 32, 64 or 128 bps. Two other tools can extract data for certain chromosome out of a BED file or a WIGGLE file. Furthermore, many Galaxy functions that we considered to be very useful for ChIP-chip/seq data analyses are also enabled in Cistrome. For example, the intersect tool for two interval files, and the filtering/sorting/cutting tool for tab-delimited text files are widely used in many of our precompiled public workflows to post-process intermediate results then feed them into downstream tools (Table S2 in Additional file 1).

## Comparison to existing software

Cistrome was built upon the Galaxy framework to provide a user-friendly, reproducible and transparent workbench for cistrome researchers. Researchers can easily and intuitively reuse and share data, incorporate published data, and publish their results on the website. Compared with the more general Galaxy main site [31], the Cistrome system was specifically designed for downstream data analysis accompanied by ChIP-chip or ChIP-seq technologies and includes basic analyses from peak calling to motif detection. In the future, the Cistrome analysis platform module will be linked to our local Data Collection (DC) module where publicly available ChIP-chip and ChIP-seq data are downloaded and preprocessed.

There are several integrative software packages designed for ChIP-chip and ChIP-seq analysis, including the widely used CisGenome platform [3] and the recently published seqMINER platform [4]. CisGenome works as a package of command line software for Linux, Windows and Mac OSX and provides a GUI and genome browser only for the Windows operating system. seqMINER works as standalone GUI software based on Java. The major difference between Cistrome and these packages is that we focus on a web solution to eliminate the trouble of maintaining various software and the demand for powerful hardware from the user. Another advantage of using a web server is that we can continue to provide Cistrome improvements, such as bug fixes and additional features, that are transparent to the user. Galaxy infrastructure enables every Cistrome tool to remember the run-time parameters in the server. When a Cistrome function is updated, users can rerun an analysis or reproduce a result using several simple mouse clicks. Last but not least, Cistrome has been provided with the workflow and data sharing features from the Galaxy framework. Users can customize their own pipeline to increase productivity. Additionally, users can share their raw data and analysis results with collaborators and the public through the web interface. An overview of a comparison of the functionalities of Cistrome, CisGenome and seqMINER is provided in Table 1 (detail in Table S3 in Additional file 1).

**Table 1:** Overview comparison of functionalities of Cistrome, CisGenome and SeqMINER

	Cistrome	CisGenome 2	SeqMINER 1.2.1
**Data preprocessing**			
ChIP-chip preprocessing	Yes. Affymetrix or NimbleGen platform	Yes. Affymetrix or other platform through conversions	Not available
ChIP-seq preprocessing	Yes	Yes. No support for SAM/BAM	Not available
General peak calling	Yes. Through wiggle file for signals	No direct solution	Not available
Cross-platform analysis	Yes. Across different ChIP-chip platforms, or across different ChIP-seq libraries	Not available	Not available
**Expression analysis**			
From normalization, differential expression, to gene ontology	Yes. Affymetrix or NimbleGen platform	Not available	Not available
**Integrative analysis**			
Genome association study	Yes. Chromosome or gene feature enrichment; aggregation plot; genes or peaks centered annotation; conservation plot; k-means clustering heatmap	Yes. Closest genes around peaks	Yes. K-means clustering at peak sites; interactive heatmap; aggregation plot
Correlation between samples	Yes. Whole genome or peak centered Pearson correlation; Venn diagram	Not available	Yes. Pearson correlation at enriched regions
Motif analysis	Yes. Find enriched known or *de novo *motifs; map motifs to genomic locations	Yes. Find *de novo *motifs; map motifs to genomic locations	Not available
**Other tools**	Liftover both BED/WIGGLE files; low level operations on text manipulation and format conversion through Galaxy	Many useful scripts for format conversions, to calculate overlaps and so on	Not available
**Genome browser visualization**	Redirect to mirrored UCSC genome browser on Cistrome, or external genome browsers supported by Galaxy	Local installed genome browser on Windows operating system	Not available

## Conclusions and future directions

We have deployed a comprehensive ChIP-chip and ChIP-seq analysis platform called Cistrome by integrating publicly available research tools and newly developed algorithms from our group under the Galaxy framework. Cistrome covers most of ChIP-chip/seq analysis tasks, from data preprocessing, expression analysis, integrative analysis, reproducible pipeline, to data publishing; this integrated approach allows biologists to analyze and visualize their own ChIP-chip/seq data for publication. We plan to extend Cistrome in the following areas: first will be to support the increasing number of ChIP-seq datasets by building a Cistrome DC module; second, we plan to continue adding additional research tools and improve the existing features to provide more sophisticated integrative workflows, especially for epigenomics data. We will address these plans in detail in the following paragraphs.

Each ChIP-chip/seq platform has its own cistrome data analysis challenges. ChIP-chip platforms include tiling arrays from Affymetrix, NimbleGen and Agilent, and ChIP-seq platforms include NGS machines from Illumina, Applied Biosciences and Helicos. A typical human ChIP-seq experiment sequenced on one Illumina GAIIx lane generates approximately 20 GB of fastq data. With more researchers adopting ChIP-chip/seq methods and NGS technologies that are improving at rates beyond Moore's law [32], the production of cistrome data is increasing exponentially. Currently, databases such as the National Center for Biotechnology Information (NCBI) Gene Expression Omnibus (GEO) [33] and the European Bioinformatics Institute (EBI) ArrayExpress [34] host array data, and databases such as the NCBI Sequence Reads Archive (SRA) [35] and the EBI SRA host sequencing data [36]. However, experimental biologists often cannot understand or reuse these deposited data in their raw form. Although some processed datasets have been submitted to these databases, they are difficult to compare and integrate due to diverse data generation platforms and analysis algorithms. Therefore, parallel to the Cistrome data analysis module, we are designing another major component of Cistrome: the DC module. The Cistrome DC will be a manually curated data warehouse. The data stored in the DC module include both raw and preprocessed data - peak locations and signal profiles - that are ready to be imported into the current Cistrome analysis platform. We plan to develop a user-friendly interface to let users easily search and browse the datasets. We also plan to build a bridge from the current analysis module to the Cistrome DC so that users can choose to package their analyzed data and publish them in the Cistrome DC upon paper publication.

Concurrent with an increasing interest in epigenomics research, increasing amounts of histone modification ChIP-seq, nucleosome-seq, and DNase-seq data are becoming available to the public. We plan to add another specific peak caller, Nucleosome Positioning from Sequencing (NPS), to Cistrome to target histone modification data [37]. When ChIP-seq data are used at the nucleosome resolution (that is, where experimentalists use micrococcal nuclease to digest DNA) NPS can provide better data interpretation than the general ChIP-seq peak caller MACS. NPS can give the well-positioned nucleosomes as output and further detect the dynamic chromatin regions with moving nucleosome or DNase sites between conditions. Our newly developed algorithms, called Binding Inference from Nucleosome Occupancy Changes (BINOCh) [38], can follow up with motif analysis in the dynamic regions to better understand the transcription factor binding changes.

Many new features and tools for cistrome analysis are included in our future plans. Basic file manipulation tools - for example, the BedTools [39] suite - will be added to Cistrome in the future. The goal is to provide more flexible workflows for different demands. Because the WIGGLE format used to save whole genome signal profiles is too big to maintain and manipulate, we plan to switch to a more space-efficient self-indexed binary format: the BigWig [40]. We also plan to support preprocessed RNA-seq data (for example, in RPKM (reads per kilobase of exon model per million mapped reads) form) in our expression analysis module. Galaxy has included Cufflinks tools in main codes, and we will provide functions that are similar to those of the current expression tools such as DESeq [41] or edgeR [42] and incorporate them into other integrative analysis tools. For example, by combining expression profiles and transcription factor motif enrichment, we could predict the correct transcription factors that collaborate with the ChIPed factor.

Because Cistrome was built on Galaxy, we will continue updating the Galaxy framework codes for new features, such as Galaxy Pages for the reproducible and interactive supplementary material or Galaxy Visualization to show data tracks in a genome browser view. We also plan to follow in the steps of Galaxy and provide a cloud computing solution for future scalability. We welcome feedback from users regarding new features and better representations to make Cistrome a better resource for the community.

## Abbreviations

bp: base pair; ChIP: chromatin immunoprecipitation; DC: Data Collection; GO: Gene Ontology; NGS: next-generation sequencing; TSS: transcription start site; TTS: transcription termination site.

## Competing interests

The authors declare that they have no competing interests.

## Authors' contributions

TL, MB, and XSL designed the project. TL, JAO, and XSL wrote the manuscript. TL, JAO, MP, MB, YT, and XSL revised the manuscript. TL, JAO, LT, CAM, BL, YZ, HGS, SSW, JM, UJP, YC, and KY implemented the system. TL, LT, and JM maintain the public server instance hosted in Dana-Farber Cancer Institute. All authors read and approved the final manuscript.

## Supplementary Material

Additional file 1 **Supplementary Tables S1, S2 and S3**. File formats and restrictions on the Cistrome server; public workflows; and detailed comparison between Cistrome and CisGenome or seqMINER. Online demonstration of a general ChIP-seq analysis can be found at the public Cistrome site [44]. Click here for file
