# Supplementary material for: Cistrome: an integrative platform for transcriptional regulation studies
Source: Genome Biol. 2011 Aug 22;12(8):R83. doi: 10.1186/gb-2011-12-8-r83 (PMC3245621; doi:10.1186/gb-2011-12-8-r83)
Supplement: Additional file 1 — Supplementary Tables S1, S2 and S3. File formats and restrictions on the Cistrome server; public workflows; and detailed comparison between Cistrome and CisGenome or seqMINER. Online demonstration of a general ChIP-seq analysis can be found at the public Cistrome site [44]. [file gb-2011-12-8-r83-S1.DOC]

Additional file 1

**Table S1 The file formats in Cistrome and restrictions of input files on current Cistrome server**

- Power users can download and install their own Cistrome instance with such restrictions removed.
- Any single output or intermediate file can’t exceed 10GBytes on current Cistrome server.

| **Format** | **Description** | **As input of** | **As output of** | **Restriction** |
| --- | --- | --- | --- | --- |
| NDF | NimbleGen array design file | MA2C |  | < 500MBytes |
| POS | NimbleGen array design file | MA2C |  | < 500MBytes |
| PairData | NimbleGen array raw probe signal file | MA2C |  | < 500MBytes |
| CEL | Affymetrix array raw probe signal file | MAT, Gene expression index |  | < 500MBytes |
| SAM | Sequence Alignment Map (capable to store pair-end sequencing data) | MACS |  | < 10GBytes |
| BAM | Compressed binary version of SAM (capable to store pair-end sequencing data) | MACS |  | < 10GBytes |
| ELAND_result | Solexa GAPipeline alignment result | MACS |  | < 10GBytes |
| ELAND_multi | GAPipeline multiple alignment result | MACS |  | < 10GBytes |
| ELAND_export | Yet another GAPipeline output for alignment | MACS |  | < 10GBytes |
| BOWTIE | Bowtie default mapping result | MACS |  | < 10GBytes |
| BED | General genomic regions | MACS, Multiple wiggle files correlation in given regions, Two wiggle file correlation in union regions, Venn diagram, SitePro, GCA, Gene2Peak, CEAS, Conservation plot, Heatmap, SeqPos, Motif Scan, Extract data from Bed | MA2C, MAT, MACS, MMChIP, Call Peaks from WIGGLE, Gene2Peak, Heatmap, Motif Scan, Extract data from Bed | < 10GBytes (MACS)  < 100K lines (Others) |
| WIGGLE | A file format for continuous data defined by UCSC genome browser. We support only the ‘variableStep’ option for WIGGLE format in most of our tools. | Multiple wiggle files correlation, Multiple wiggle files correlation in given regions, Two wiggle file correlation in union regions, Call Peaks from WIGGLE, SitePro, Liftover Wig Files, Standardize wig file, Extract data from Wiggle | MA2C, MAT, MACS, MMChIP, Liftover Wig Files, Standardize wig file, Extract data from Wiggle | < 2GBytes |
| PDF | Portable Document Format created by Adobe |  | Multiple wiggle files correlation, Multiple wiggle files correlation in given regions, CEAS, SitePro, Gene expression index, Draw a histogram/box plot tool ( expression ) |  |
| PNG | Portable Network Graphics format |  | Multiple wiggle files correlation, Multiple wiggle files correlation in given regions, Two wiggle file correlation in union regions, Venn Diagram, Conservation plot, Heatmap |  |
| CEL.zip | A zip file containing at least two Affymetrix CEL files for expression microarray, plus an optional .TXT pheno file. | Gene Expression Index | Expression CEL file packager | No restriction. |
| XYS.zip | A zip file containing at least two NimbleGen XYS files for expression microarray, plus an optional .TXT pheno file. | Gene Expression Index |  | No restriction. |
| Expression index file in text format | It contains sample names in the columns and Gene symbols in the rows | Calculate differential expression, Calculate highest expressed TFs, Find correlated genes or TFs, Draw a histogram/box plot of expression index | Gene Expression Index | No restriction. |
| Differential gene list in text format | It contains ‘Gene ID’, ‘Log2 ratio’ and ‘P value’ columns | Conduct GO | Calculate differential expression | No restriction. |
| Motif xml file | An output from SeqPos algorithm, containing de novo motif PSSMs. | Motif Scan | SeqPos | No restriction. |
| HTML file | An output from SeqPos algorithm, providing a sortable list of enriched motifs, motif logos and motif annotations. |  | SeqPos | No restriction. |

**Table S2. The public workflows for ChIP-chip/seq analysis**

| **Name** | **Description** | **Tools involved** |
| --- | --- | --- |
| Demo ChIP-chip on Affymetrix Tiling Array | A demo ChIP-chip pipeline for Affymetrix human tiling array version 2 (hg18 assembly) of single replicate | MAT, Gene2Peak, CEAS, SeqPos, Conservation plot, Galaxy: Convert whitespace to tab, Sort, Select first |
| General ChIP-chip on NimbleGen Tiling Array | A generic ChIP-chip pipeline for NimbleGen tiling array of single replicate | MA2C, Gene2Peak, CEAS, SeqPos, Conservation plot, Galaxy: Convert whitespace to tab, Sort, Select first |
| General ChIP-seq | A generic ChIP-seq pipeline for Next Generation Sequencing platform data of single replicate | MACS, Gene2Peak, CEAS, SeqPos, Conservation plot, Galaxy: Convert whitespace to tab, Sort, Select first |
| ChIP-seq with two replicates | Calculate correlation of two ChIP-seq replicates | MACS, Multiple wiggle files correlation, Two wiggle file correlation in union regions, Venn diagram |
| Generate differential gene list | Take the differential expression result and generate the up/down-regulated genes, which can be used in CEAS. | Galaxy: Convert whitespace to tab, Remove beginning, Filter, Cut |
| From Heatmap clustering to Gene names | Take the Heatmap clustering results on gene TSSs, then separate the first 5 clusters with distinct patterns, which can be followed by GO analysis | Galaxy: Remove beginning, Filter, Cut |
| BAM to BED | Convert BAM format file to BED while filtering out unmapped reads | Galaxy: BAM to SAM, Filter SAM, Convert SAM to intervals, Convert intervals to BED |
| Randomly select reads in BAM | Randomly sample BAM file to given number of reads in BED format | Galaxy: BAM to SAM, Filter SAM, Select random lines, Convert SAM to intervals, Convert intervals to BED |
| Find regions with two different motifs | Scan given regions of two different motifs, find the regions with two non-overlapping different motifs | SeqPos, Galaxy: Intersect, Substract |

**Table S3 Compare Cistrome functions to CisGenome and seqMINER**

| **Cistrome features** | **Description** | **CisGenome comparison** | **seqMINER comparison** |
| --- | --- | --- | --- |
| **Import Data** |  |  |  |
| Data upload ( modified Galaxy function ) | Directly upload through web page or HTTP/FTP external links. Cistrome adds gene expression ZIP file supports to Galaxy general upload tool. | Load local file from user’s computer. It doesn’t support expression data. | Load local file from user’s computer. It doesn’t support expression data. |
| Expression data packager | Retrieve CEL files directly from GEO FTP and package them in a zip file for gene expression analysis. | Not available | Not available |
| **Peak Calling** |  |  |  |
| ChIP-chip analysis on Affymetrix array | MAT algorithm for Affymetrix promoter or whole genome tiling arrays. | TileMap for Affymetrix tiling arrays. | Not available |
| ChIP-chp analysis on NimbleGen array | MA2C algorithm for NimbleGen tiling arrays. | TileMap. Special conversion is needed. | Not available |
| ChIP-seq analysis | Recent version of MACS algorithm. Support SAM/BAM/BED/ELAND format input files with or without control. | SeqPeak. Multiple steps to convert, call peaks, FDR calculations, with or without control. Not support BAM/SAM format. | Not available |
| General peak caller | Normalize any source of signal profile in WIGGLE format then use z-scores to call enriched regions. | No direct solution. May be implemented with multiple conversions on inputs. | Not available |
| Meta analysis of ChIP-chip | Combine the signals from different array platform or design, use meta-analysis to call enriched regions. Based on MM-ChIP algorithm. | Not available | Not available |
| Meta analysis of ChIP-seq | Combine different libraries with different fragment sizes, and use a MACS-like algorithm to find the overall enriched regions. | Not available | Not available |
| **Genome association study** |  |  |  |
| Enrichment on chromosome, gene annotations. Aggregation plots on TSS/TTS and meta-gene body. | Use CEAS main program to generate PDF or PNG report in separate pages. Can incorporate gene expression information. | Need multiple scripts and a careful design to perform the same functionality. | Not available |
| Aggregation plots centered at given genomic regions | Use SitePro program; Multiple region sets or multiple signal profiles are allowed. | Not available | Aggregation plots can only be drawn after the clustering. Not support multiple signal profiles. |
| Gene centered annotation | Use GCA program; Find the binding sites near genes; Calculate the coverage of the enriched regions at the gene body. | Multiple scripts should be combined. | Not available |
| Peak centered annotation | Use Peak2Gene program; Find the genes near binding sites with certain cutoff. | Multiple scripts should be combined. | Not available |
| Conservation analysis | Draw an average conservation plot around given genomic locations. | Multiple scripts should be used to extract the conservation scores around given regions and summarize. | Not available |
| Heatmap with clustering | K-means clustering based on signals around given locations; Draw heatmap with customizable color schema. | Not available | K-means clustering has more normalization methods. Heatmap is interactive.­ |
| **Correlation** |  |  |  |
| Correlation between different signal profiles in whole genome scale | Pearson correlation coefficients are calculated; scatterplot or heatmap is provided. | Not available | Not available |
| Correlation between different signal profiles in a genomic location set | Pearson correlation coefficients are calculated; scatterplot or heatmap is provided. | Not available | Similar to Cistrome. |
| Correlation for two signal profiles in the union regions from two peak files | Pearson correlation coefficients are calculated. It’s better to calculate correlation of two replicates. | Not available | Not available |
| Venn diagram | Calculate the overlap between two or three sets of genomic locations and draw a venn diagram using Google Chart API. | Not available | Not available |
| **Expression** |  |  |  |
| Gene expression normalization | Use RMA/GCRMA/JustRMA/MAS5 in bioconductor/R; use customized CDFs from BRAINARRAY; support Affymetrix and NimbleGen gene arrays. | Not available | Not available |
| Differential expression analysis | Use LIMMA in bioconductor/R. | Not available | Not available |
| Find highest expressed TFs | Use Gene Ontology terms to filter the highly expressed transcription factors. | Not available | Not available |
| Find correlated genes or TFs | Use correlation or GO terms to find a subset of genes from a given gene. | Not available | Not available |
| GO analysis | Use GOstats in bioconductor/R and a remote call to DAVID. | Not available | Not available |
| Histogram/boxplot comparing expression of different gene groups | Compare the gene expression level for a given list of genes in different conditions. | Not available | Not available |
| **Motif analysis** |  |  |  |
| Find the enriched motifs from given locations | Use the SeqPos algorithm; Both de novo motif discovery and a known motif scan in five motif database; optimize the distance from motif to the centers of give locations. | Only de novo motif discovery; multiple scripts needed. | Not available |
| Motif scan | Find the occurrence of a given motif in a given set of regions. | Similar to Cistrome; multiple scripts needed. | Not available |
| **Liftover/Other** |  |  |  |
| Convert signal profile from one genome assembly to another | Liftover the signal profile in wiggle format from one assembly to another. | Not available | Not available |
| Convert peak regions from one genome assembly to another ( Galaxy function ) | Liftover the peak regions in BED format from one assembly to another. Implemented in Galaxy framework. | Not available | Not available |
| Convert signal profile with specific resolution | Standardize signal file in wiggle format by converting solution to 8, 32, 64, or 128 bps. | Not available | Not available |
| Extract data from signal profile for a chromosome | Extract data from signal profile in wiggle format for a given chromosome. | Not available | Not available |
| Extract data from peak regions file for a chromosome | Extract data from peak regions in BED format for a given chromosome. | Not available | Not available |
| **Low level operations** | Fetch annotations from public databases; text manipulation, extract sequences, sort/filter tab-delimited files, convert formats and so on, borrowed from Galaxy. | Many useful scripts for the same functions. | Not available |
| **Visualization on genome browser** | Redirect to Galaxy supported genome browsers or UCSC local mirror on Cistrome. | A local genome browser server should be installed in Windows OS. | Not available |
| **Data sharing and publishing** | Provided by Galaxy infrastructure. | Not available | Not available |
| **Workflow for one-click solutions** | Create and share workflow for reproducible or repetitive analysis; provided by Galaxy infrastructure. | Not available | Not available |
